# Worsening of Preexisting Psychiatric Conditions During the COVID-19 Pandemic

**DOI:** 10.3389/fpsyt.2020.581426

**Published:** 2020-12-16

**Authors:** Susanna Gobbi, Martyna Beata Płomecka, Zainab Ashraf, Piotr Radziński, Rachael Neckels, Samuel Lazzeri, Alisa Dedić, Asja Bakalović, Lejla Hrustić, Beata Skórko, Sarvin Es haghi, Kristina Almazidou, Luis Rodríguez-Pino, A. Beyza Alp, Hafsa Jabeen, Verena Waller, Dana Shibli, Mehdi A. Behnam, Ahmed Hussain Arshad, Zofia Barańczuk-Turska, Zeeshan Haq, Salah U. Qureshi, Ali Jawaid

**Affiliations:** ^1^Zurich Center for Neuroeconomics, University of Zurich, Zurich, Switzerland; ^2^Methods of Plasticity Research, Department of Psychology, University of Zurich, Zurich, Switzerland; ^3^Faculty of Arts, University of Waterloo, Waterloo, ON, Canada; ^4^Faculty of Mathematics, Informatics and Mechanics, University of Warsaw, Warsaw, Poland; ^5^Biomolecular Sciences Graduate Program, Department of Biomolecular Sciences, Boise State University, Boise, ID, United States; ^6^Faculty of Science and Engineering, University of Groningen, Groningen, Netherlands; ^7^Faculty of Medicine, University of Tuzla, Tuzla, Bosnia and Herzegovina; ^8^Faculty of Medicine, Medical University of Warsaw, Warsaw, Poland; ^9^Faculty of Medicine, Shahid Beheshti University of Medical Sciences, Tehran, Iran; ^10^Faculty of Veterinary Medicine, Aristotle University of Thessaloniki, Thessaloniki, Greece; ^11^Faculty of Medicine, University of Valencia, Valencia, Spain; ^12^Faculty of Medicine, Maltepe University, Istanbul, Turkey; ^13^Medical College, Dow University of Health Sciences, Karachi, Pakistan; ^14^Department of Radiation Oncology, University Hospital Zurich, University of Zurich, Zurich, Switzerland; ^15^Faculty of Medicine, University of Jordan, Amman, Jordan; ^16^Neuroscience Center Zurich, University of Zurich/Swiss Federal Institute of Technology (ETH), Zurich, Switzerland; ^17^Baqai Medical University, Karachi, Pakistan; ^18^Institute of Mathematics, University of Zurich, Zurich, Switzerland; ^19^Texas Behavioral Health, Houston, TX, United States; ^20^BRAINCITY Center of Excellence for Neural Plasticity and Brain Disorders, Nencki Institute of Experimental Biology, Warsaw, Poland; ^21^Department of Neurology, University of Texas Health Science Center, Houston, TX, United States

**Keywords:** COVID-19, psychiatric patients, worsening, depression, post traumatic stress disorder, general psychological disturbance

## Abstract

**Objectives:** To ascertain factors associated with worsening of psychiatric conditions during the coronavirus disease 2019 (COVID-19) pandemic.

**Methods:** This study anonymously examined 2,734 psychiatric patients worldwide for worsening of their preexisting psychiatric conditions during the COVID-19 pandemic. An independent clinical investigation of 318 psychiatric patients from United States was used for verification.

**Results:** Valid responses mainly from 12 featured countries indicated self-reported worsening of psychiatric conditions in two-thirds of the patients assessed that was through their significantly higher scores on scales for general psychological disturbance, posttraumatic stress disorder, and depression. Female gender, feeling no control of the situation, reporting dissatisfaction with the response of the state during the COVID-19 pandemic, and reduced interaction with family and friends increased the worsening of preexisting psychiatric conditions, whereas optimism, ability to share concerns with family and friends, and using social media like usual were associated with less worsening. An independent clinical investigation from the United States confirmed worsening of psychiatric conditions during the COVID-19 pandemic based on identification of new symptoms that necessitated clinical interventions such as dose adjustment or starting new medications in more than half of the patients.

**Conclusions:** More than half of the patients are experiencing worsening of their psychiatric conditions during the COVID-19 pandemic.

Coronavirus disease 2019 (COVID-19) has emerged as the most critical global crisis of the 21st century. COVID-19 cases have exceeded 30 million as of September 28, 2020 (1). A number of studies have indicated an increase in anxiety, depression, and other psychopathologies during the COVID-19 pandemic (2–7). Such disturbances have occurred, in particular, in individuals with previous history of psychological illness, who have also been found to be at increased risk of contracting COVID-19 (8). Furthermore, worsening of psychiatric conditions has also been associated with higher risk of suicidal ideation (9, 10).

Psychiatric conditions constitute a significant burden on healthcare systems and economy (4, 11–13). The COVID-19 pandemic has given rise to even greater challenges for an already struggling system of mental health care. Moreover, it has been associated with an increase in use of mental health and suicide prevention helplines (14). Furthermore, new methods of psychological/psychiatric care delivery through telemedicine are being increasingly adopted (15). It is paramount for the optimization of mental healthcare delivery during these challenging times that the most vulnerable populations are efficiently identified.

To address this, we analyzed the data from participants with preexisting psychiatric conditions from our global study on the mental health impact of COVID-19 (10). Each patient report of worsening of psychiatric conditions was then cross-analyzed with participants' demographics, opinions/outlooks, personality traits, current household conditions, previous history, and other factors associated with COVID-19 to identify risk and resilience factors for worsening psychiatric condition. The results were then verified in an independent clinical cohort of psychiatric patients that consulted a psychiatry practice in Houston, TX, USA, during the COVID-19 pandemic.

## Methods

### Study Design

The study comprised two independent evaluations: (1) a cross-sectional electronic survey-based assessment of individuals older than 18 years willing to participate in the study and (2) evaluation of anonymized clinical records of psychiatric patients older than 18 years.

### Online Survey

The anonymous online survey was conducted among participants from diverse demographic groups to examine the status of their preexisting psychiatric conditions that were verified via standardized self-report scales for general psychological disturbance, risk for posttraumatic stress disorder (PTSD), symptoms of depression, and suicidal ideation. The survey was available online for a period of 15 consecutive days beginning 18:00 Central European Time on March 29, 2020, and concluding on 18:00 Central European Time on April 14, 2020.

The questionnaire was developed through close consultation between a neuroscientist, a neuropsychologist, a psychiatrist, a data scientist, and a psychiatry clinic manager. The questionnaire included closed-ended questions that assessed participant characteristics and opinions and screened for neuropsychiatric symptoms through standardized and validated self-report scales. The questionnaire prototype was prepared in English (Appendix 1) and translated into 10 additional languages (Arabic, Bosnian, French, German, Greek, Italian, Persian, Polish, Spanish, and Turkish; Appendix 2). The translation was performed by bilingual native speakers and vetted by volunteers native to those countries. The feasibility of each questionnaire was confirmed using pilot studies that comprised 10 participants each. These responses were excluded from the final analysis.

The questionnaires (Appendix 1) included a section on participant demographics (age, gender, country, residential setting, educational status, current employment status), household conditions (working/studying from home, home isolation conditions, pet ownership, level of social contact, social media usage, time spent exercising), COVID-19–related factors (knowing a coworker, friend, or family member who tested positive for or demised due to COVID-19; prediction about pandemic resolution), personality traits (level of optimism, level of extroversion), previous history of psychiatric disease and/or trauma, previous exposure to human crisis, and level of satisfaction with actions of the state and employer during the current crisis. All questionnaires were rated on binary (yes/no) responses or Likert-type scales.

The other sections contained general health assessment based on World Health Organization (WHO) Self-Reporting Questionnaire-20 (SRQ), Impact of Event Scale (IES), and Beck's Depression Inventory II (BDI) (16–18). These scales were chosen based on their usage and efficacy in previously employed works studying the psychological impact of human crises such as the severe acute respiratory syndrome epidemic (19–27). IES wording was purposefully adjusted to assess the impact of an ongoing event rather than a past event.

Using a nonrandomized referral sampling (snowball sampling) method, participants were contacted by a team of 70 members (study authors and volunteers who have been acknowledged in the *Acknowledgments*) using electronic communication channels including posts on social media platforms, direct digital messaging, and personal and professional email lists. For the survey, 12 countries were included in the “featured” list. These countries included United States, Spain, Italy, France, Germany, Iran, Turkey, Switzerland, Canada, Poland, Bosnia and Herzegovina, and Pakistan. The data collection procedures were repeated at least thrice during the data collection period (March 29–April 14, 2020).

An overall total of 13,332 responses were collected. Surveys were excluded if they were completed by participants who were younger than 18 years (*n* = 34), were missing responses for all dependent variables (*n* = 112), had been submitted previously (*n* = 325), were missing geographic location (*n* = 20), or were from WHO AFRO region (*n* = 24). When the responses were missing for individual items, the missing data were considered null and excluded from the analysis for that particular variable. In this follow-up study, however, only responses from participants who reported suffering from a previous psychiatric condition (*n* = 2,734) were considered valid.

### Clinical Study

The clinical data were extracted and analyzed for all the adult patients who consulted for online follow-up clinical evaluations at a psychiatric care facility (Texas Behavioral Health) based in Houston, TX, USA, during March 29–April 14, 2020. The inclusion criteria were previous diagnosis of major depressive disorder or anxiety disorders (generalized anxiety disorder and PTSD). Patients with diagnoses other than these and those younger than 18 years were excluded. Only the data from patients consenting to use of their records for this research were included in the study.

Clinical data for each patient were examined by clinic assistants blinded to the study design. The following information was extracted: age, gender, home-isolation status during COVID-19, social support during COVID-19, past exposure to trauma or a human crisis situation, and clinical diagnosis. Worsening of psychiatric conditions was assessed based on clinician report of new symptoms, need to increase or adjust the medication, and referral for a new therapy.

Data from 318 patients were considered valid for analysis. When the responses were missing for individual items, the missing data were considered null and excluded from the analysis for that particular variable.

### Ethical Considerations

Informed consent was obtained from each survey and clinical participant to allow anonymous recording, analysis, and publication of their answers. The data were collected in a completely anonymous fashion without recording any personal identifiers, and the confidentiality of the participants was maintained throughout all phases of the study. The study procedures were reviewed and approved by the University of Zurich Research Office for Scientific Integrity and Cantonal Ethics Commission for the canton of Zurich (Switzerland), BRAINCITY, Nencki Institute of Experimental Biology, Warsaw (Poland), and the Faculty of Medicine, University of Tuzla, Tuzla (Bosnia and Herzegovina).

### Statistical Analysis

All statistical analyses were performed using R version v.3.6.3 and *Rstudio* (28). All figures were produced using the packages *ggplot2* (29) and *CGPfunctions* (30)

Unadjusted analysis for worsening of psychiatric conditions in both the survey and the clinical cohort involved Fisher exact test. For the survey, the categorical predictors included gender, residential status, education level, employment status, being a medical professional, working remotely from home, satisfaction with employer, satisfaction with the state (government), home-isolation status, interaction with family and friends, social media usage, ability to share concerns with a mental health professional, ability to share concerns with family and friends, prior exposure to a human crisis situation, previous exposure to trauma, level of extroversion, prediction about COVID-19 resolution, and one's self-determined role in the pandemic. For the clinical study, the categorical predictors included age, gender, home isolation status, and social support during home isolation.

Multiple logistic regression models were built to generate odds ratios (ORs) for worsening of psychiatric conditions both in the survey and the clinical cohorts. All statistical analyses were performed by the analysis team comprising MP, SG, PR, and AJ in consultation with ZB.

## Results

### Survey Study

#### Demographics

**Figure 1:**
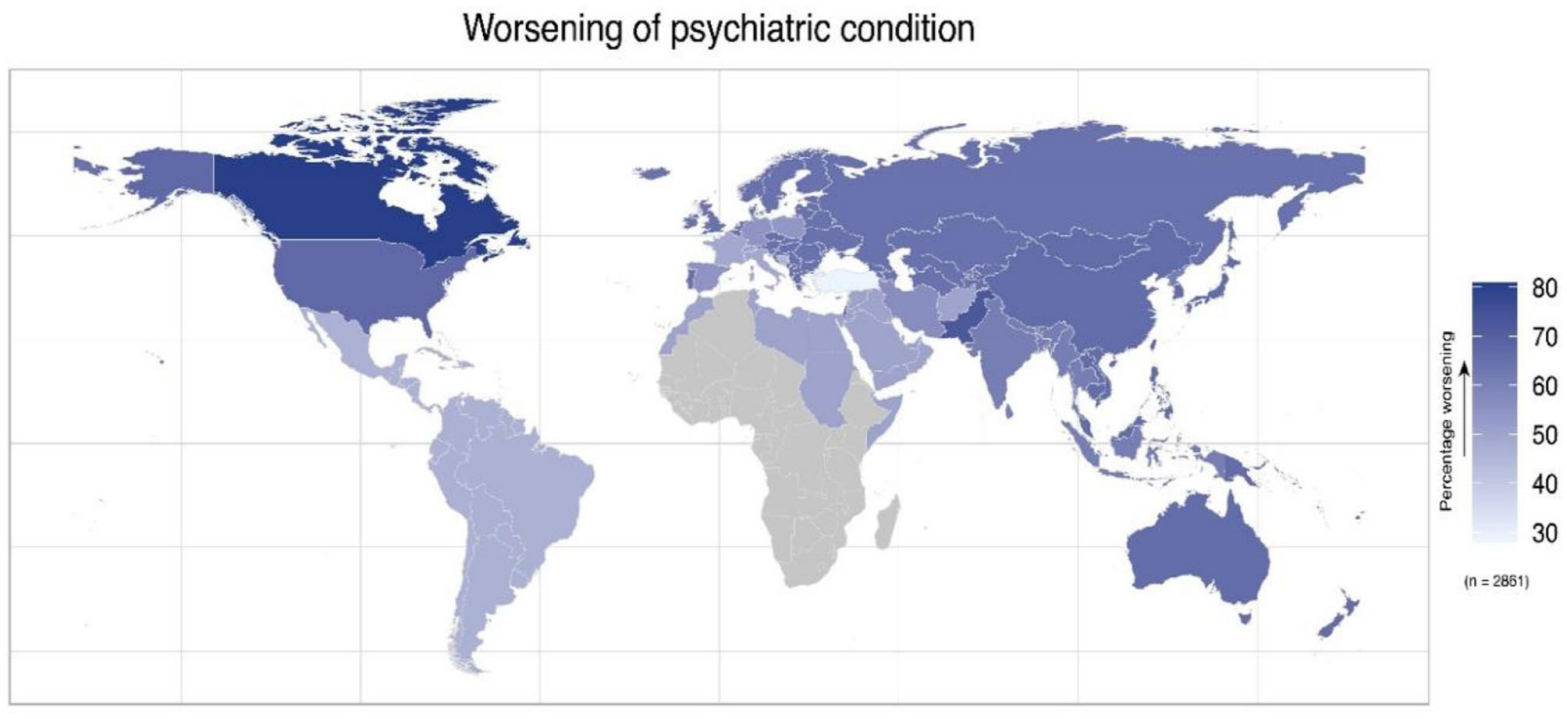
Geodemographic representation of the survey participants with preexisting psychiatric condition that reported worsening of their condition. The map shows the percentage of worsening preexisting psychiatric conditions separately for each of the featured countries and for each of WHO regions.

A total of 2,734 responses were considered valid with the highest responses from the United States (874), Poland (255), Canada (246), Spain (205), and Pakistan (203). The distribution of the responses across the 12 featured countries and the WHO regions is presented in Supplementary Item 1. Canada had the highest (80.89%) proportion of patients reporting worsening of their psychiatric condition followed by Pakistan (72.41%) and the United States (67.5%). Turkey had the lowest percentage (28.57%) for worsening of psychiatric conditions (Figure 1).

There was a disproportion in valid responses, with higher numbers from those participants who were female (79.44%); residing in urban areas (84.6%); with advanced educational qualification, i.e., bachelor's degree or higher (71.5%); working/studying remotely from home (65%); and currently under home isolation with a partner/family (82.77%). Also notable were responses expressing some level of satisfaction with COVID-19–related employer (52.67%) and state response (64.26%) and spending <15 min on daily physical exercise (52.99%). A majority of participants also reported increased social media usage (65.42%), less-than-usual or minimal interaction with family and friends (64.88%), and feeling some level of control in protecting themselves and others during the COVID-19 pandemic (94.36%).

**Figure 2:**
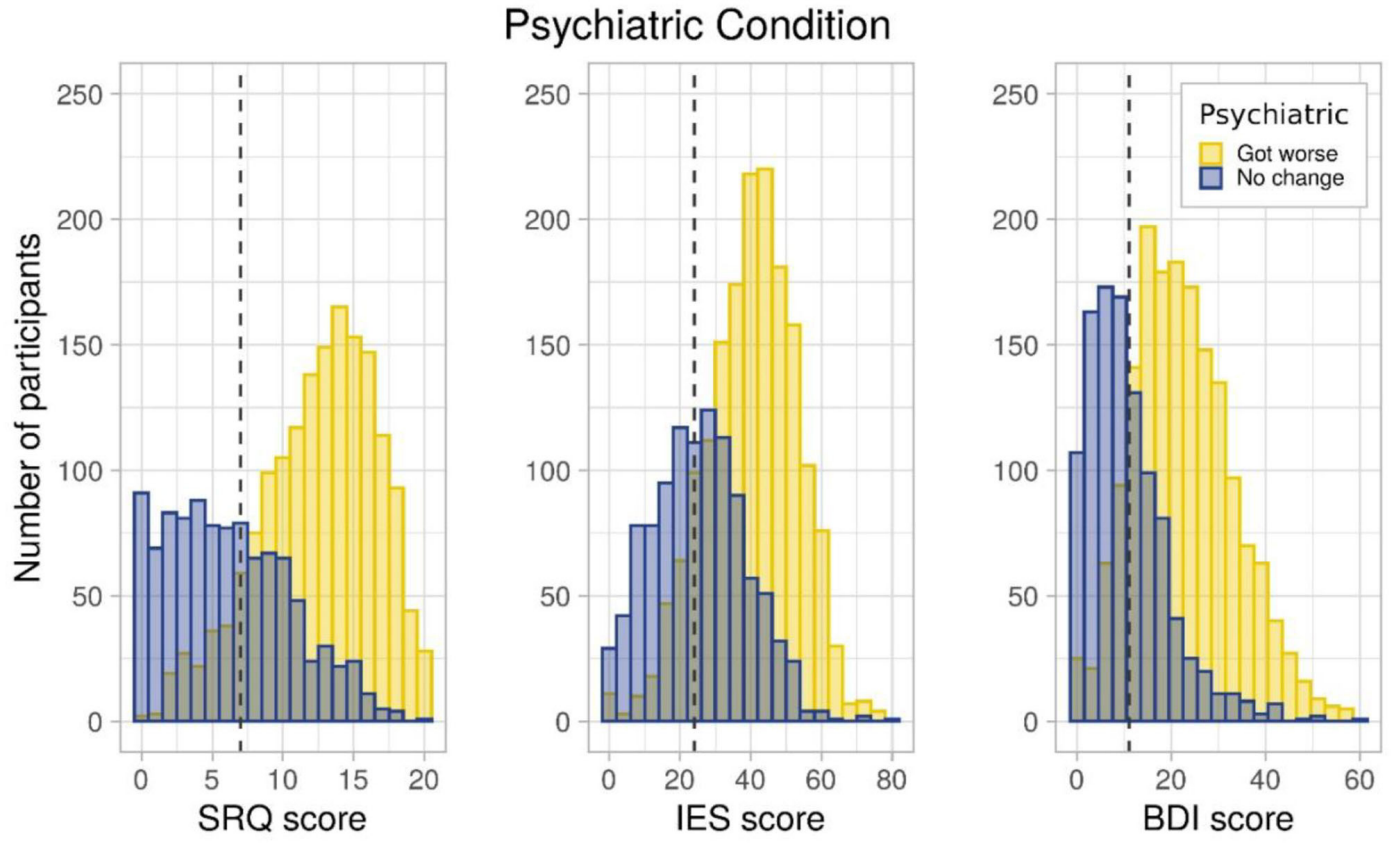
Population distribution of people with preexisting psychiatric condition across SRQ, IES, and BDI score.

Participants' report of worsening of psychiatric conditions was verified by comparing the SRQ, IES, and BDI scores between patients reporting worsening of psychiatric conditioning vs. those reporting no change. All scores were significantly (*p* < 0.05) higher in patients reporting worsening of psychiatric conditions. Distribution of patients reporting no change in their condition in comparison to worsening along the SRQ, IES, and BDI scales further confirmed this pattern (Figure 2).

#### Unadjusted Analysis of the Worsening of Psychiatric Condition

Unadjusted χ^2^ analysis of association between different patient factors and their report of psychiatric condition worsening revealed significantly higher reports of worsening in women, patients with advanced education, patients who reported being home isolated, and those with previous trauma exposure. Moreover, patients reporting dissatisfaction with the response of their government and employer during COVID-19 were more likely to report worsening of psychiatric condition. Finally, patients who identified themselves as a pessimist, felt lack of control during the current situation, and had negative prediction about COVID-19 resolution were more likely to report worsening of their psychiatric condition.

On the contrary, patients who were able to interact and share concerns with their family and friends or with a health professional like usual during COVID-19 were less like to report worsening of their preexisting psychiatric conditions. The details of the unadjusted categorical analysis are present in Table 1.

**Table 1:** Association of psychiatric condition worsening and patient demographics/characteristics.

**Predictors**		**Psychiatric condition**	
		**No change**	**Got worse**
Gender	Male (*n* = 491)	**51%**	**49%**
	Female (*n* = 2,172)	**36%**	**64%**
	Non-binary (*n* = 60)	**17%**	**83%**
	Not disclosed (*n* = 21)	**57%**	**43%**
Residence	Rural (*n* = 405)	38%	62%
	Urban (*n* = 2,314)	38%	62%
Education	Compulsory (*n* = 768)	**41%**	**59%**
	Advanced (*n* = 1,955)	**37%**	**63%**
Work status	Private employed (*n* = 521)	41%	59%
	Public employed (*n* = 604)	35%	65%
	Freelancer (*n* = 201)	38%	62%
	Unemployed (*n* = 691)	35%	65%
Medical or healthcare professional	No (*n* = 2,538)	38%	62%
	Yes (*n* = 203)	38%	62%
Remotely working from home	No (*n* = 962)	40%	60%
	Yes (*n* = 1,778)	37%	63%
Opinion about employer response to COVID-19	Not satisfied (*n* = 333)	**26%**	**74%**
	Somewhat satisfied (*n* = 554)	**32%**	**68%**
	Satisfied (*n* = 886)	**44%**	**56%**
Opinion about state response to COVID-19	Not satisfied (*n* = 983)	**32%**	**68%**
	Somewhat satisfied/ Satisfied (*n* = 1,757)	**42%**	**58%**
Home isolation	Not isolated (*n* = 169)	**50%**	**50%**
	Individual home isolation (*n* = 314)	**38%**	**62%**
	Home isolation with family or partner (*n* = 2,263)	**38%**	**62%**
Presence of pet at home	No pet at home (*n* = 1,380)	**41%**	**59%**
	Pet at home (*n* = 1,357)	**35%**	**65%**
Interaction with family or friends	Less than usual/ Minimal interaction (*n* = 1,774)	**33%**	**67%**
	Like usual (*n* = 916)	**48%**	**52%**
Use of social media	Less than usual (*n* = 195)	**31%**	**69%**
	Like usual (*n* = 759)	**53%**	**47%**
	More than usual (*n* = 1,789)	**33%**	**67%**
Time dedicated to physical exercise	<15 min (*n* = 1,449)	37%	63%
	More than 15 min (*n* = 964)	39%	61%
	More than 1 h (*n* = 328)	42%	58%
Close person positive for COVID-19	No (*n* = 2,011)	39%	61%
	Yes (*n* = 730)	37%	63%
Close person demised due to COVID-19	No (*n* = 2,562)	38%	62%
	Yes (*n* = 182)	40%	60%
Ability to share concerns with health professional	No (*n* = 1,425)	**33%**	**67%**
	Yes (*n* = 1,133)	**41%**	**59%**
Ability to share concerns with family or friends	No (*n* = 323)	**23%**	**77%**
	Less than usual (*n* = 832)	**21%**	**79%**
	Like usual (*n* = 1,589)	**51%**	**49%**
Previous exposure to crisis	No (*n* = 1,977)	38%	62%
	Yes (*n* = 762)	40%	60%
Previous exposure to traumatic experiences	No (*n* = 853)	**43%**	**57%**
	Yes (*n* = 1,426)	**35%**	**65%**
	Yes, before the age of 17 (*n* = 467)	**40%**	**60%**
Personality	Extrovert (*n* = 908)	41%	59%
	Introvert (*n* = 1,682)	37%	63%
Personality	Pessimist (*n* = 685)	**25%**	**75%**
	Optimist (*n* = 798)	**49%**	**51%**
	Realist (*n* = 1,253)	**39%**	**61%**
Prediction about COVID-19 outcome/resolution	It might be the end of human race (*n* = 46)	**13%**	**87%**
	It will resolve after many months or years (*n* = 1,037)	**37%**	**63%**
	It will resolve in the summer but not within a month (*n* = 1,457)	**39%**	**61%**
	It will resolve within a month (*n* = 159)	**43%**	**57%**
Self-opinion in COVID-19 pandemic	It is not in my control at all (*n* = 157)	**18%**	**82%**
	Still some kind of control to protect myself/others (*n* = 2,580)	**39%**	**61%**

*This table shows the percentage of participants with and without a worsening or their psychiatric condition divided according to their demographics/personal traits. The values are compared through an unadjusted χ^2^ test, and significant differences (p < 0.05) are highlighted in bold font. Specifically, each bold association indicates a difference in categories reported in the predictors' column vertically*.

#### Adjusted Analysis of the Worsening of the Psychiatric Condition

**Figure 3:**
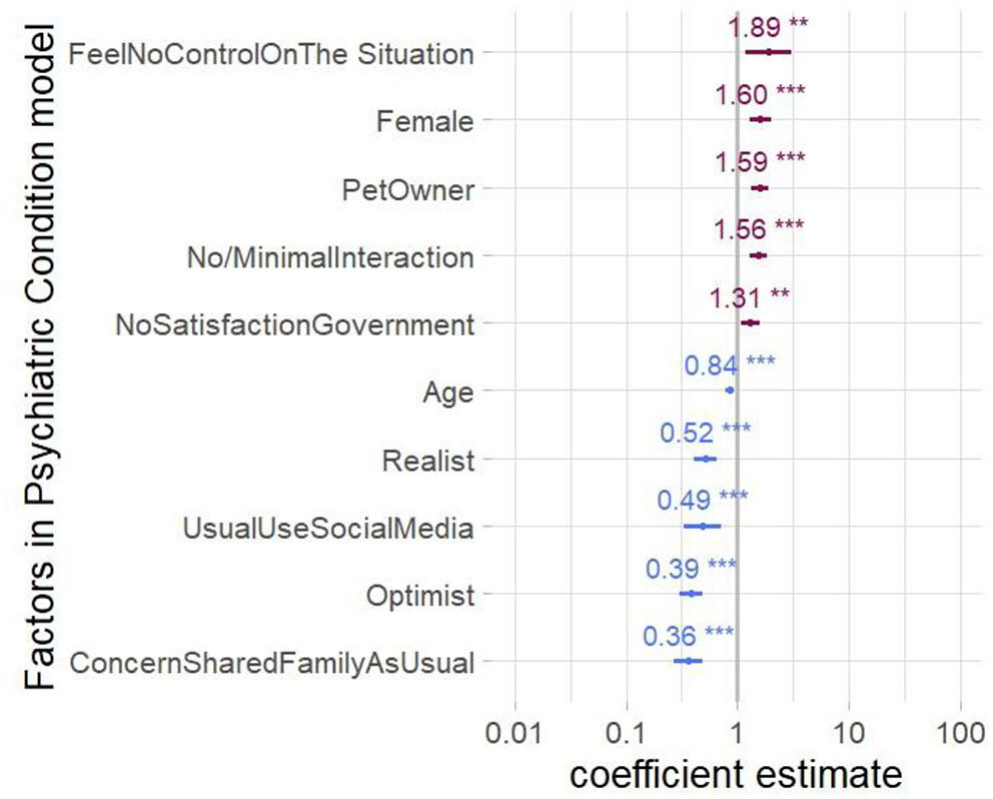
Factors associated with psychiatric condition worsening. Foster plot shows the mean estimates and the 95% confidence intervals (CIs) for adjusted coefficients significantly affecting the reported worsening of psychiatric condition by the patients. Factors indicating more odds of worsening are shown in red, whereas factors indicating less odds of worsening are in blue.

Adjusted analysis was then performed for patients' report of psychiatric condition worsening via logistic regression to adjust for confounding associations. Report of feeling no control over the situation during the COVID-19 pandemic showed an 89% increase in the odds of reporting worsening of psychiatric condition (OR, 1.89; 95% CI, 1.18–3.03). Similarly, no or minimal social interaction during COVID-19 was associated with higher odds of reporting worsening of the psychiatric condition during COVID-19 (OR, 1.56; 95% CI, 1.30–1.87). Not being satisfied with the government's response also showed an increased probability of worsening of psychiatric condition during COVID-19 (OR, 1.31; 95% CI, 1.09–1.58). Finally, female psychiatric patients were more likely to report worsening of their psychiatric condition compared to male patients (OR, 1.70; 95% CI, 1.28–2.00; Figure 3).

On the contrary, the ability to share concerns with family and friends like usual and optimistic attitude decreased the worsening of the psychiatric condition [OR, 0.39 (95% CI, 0.30–0.49) and OR, 0.36 (95% CI, 0.27–0.49)]. Furthermore, as-usual usage of social media during COVID-19 and considering oneself a realist also decreased the probability of worsening of psychiatric condition [OR, 0.49 (95% CI, 0.34–0.71) and 0.52 (95% CI, 0.41–0.65)].

### Clinical Study

The valid clinical samples comprised 71.58% females, and the diagnosis of a vast majority (83.56%) of patients was major depressive disorder. Clinicians identified new symptoms in 44% of patients, with sleep disturbance being the most common emerging symptom. Collectively, clinicians felt the need to make treatment adjustments in almost half of the patients in the form of starting a new medication or treatment modality or adjusting the dose of a currently prescribed medication (Supplementary Item 3).

Among the patient-related factors, female gender significantly increased the likelihood of a change of medication by the clinician (OR, 2.22; 95% CI, 1.03–4.49). However, other patient-related factors such as age and level of social support during home isolation were not associated with any clinical intervention (Table 2).

**Table 2:** Factors associated with clinician change of medication.

	**Change of medication**
Female	2.22^*^ (1.03–4.79)
Social Support	1.24 (0.57–2.70)
Age	0.90 (0.66–1.21)
*N*	291

* *p < 0.05*.

*Logistic regression analysis to assess the effect of patient gender, social support during home isolation, and age predicts increased likelihood of medication change by the clinician for female psychiatric patients during the COVID-19 pandemic*.

## Discussion

This study highlights a significant impact of the COVID-19 pandemic on psychiatric patients worldwide. At least 50% of the psychiatric patients evaluated in this study from 8 of the 12 featured countries reported worsening of psychiatric conditions. Notably, the self-reported worsening of psychiatric conditions was cross-validated with patients' scores on scales assessing general psychological disturbance, risk of PTSD, and depression. Severity of psychopathology assessed through these scales confirmed the patients' report of psychiatric condition worsening. Finally, clinician reports from an independent cohort of psychiatric patients in the United States confirmed that almost half of the patients reported new symptoms and required treatment adjustments during the COVID-19 pandemic.

In addition to ascertaining if there has been a general worsening of psychiatric conditions during COVID-19, a major aim of this study was to identify risk factors for such worsening. Female gender, having no or minimal interaction with family and friends, not being satisfied with the actions of the government, and feeling lack of control over the situation were associated with worsening of psychiatric conditions in the survey cohort. Patients who were older, considered themselves optimists or realists, used social media like usual, and were able share their concerns with family and friends during COVID-19 like usual were less likely to report worsening of psychiatric conditions. Notably, examination of the clinical cohort confirmed some of these findings. Clinicians reported significantly higher adjustment of medications for female psychiatric patients.

The results of this study confirm previous speculations and concerns about the vulnerability of psychiatric patients during the COVID-19 crisis (7, 31). Compared with previous studies on the impact of mental health during pandemic, this study focuses on the deterioration of psychiatric illnesses in response to COVID-19. Other studies have focused on vulnerable populations including indigenous, migrant, and imprisoned populations; people with disabilities; women (32); frontline workers (33); and the elderly (11), but thus far has not included populations with preexisting psychiatric illnesses. Tracing the worsening of psychiatric illnesses in response to COVID-19 can provide the insight necessary to improve mental health systems. Moreover, keeping the vulnerability of those with preexisting psychiatric illness in mind, health systems can become better equipped to address the concerns of this population, mitigate the risk of further mental deterioration, and reduce prevalence of suicidal ideation. Previous studies have reported the importance of adequate procedures to detect early mental health worsening (34), but have scarcely been conducted in the context of pandemics such as COVID-19.

Identifying factors that are associated with worsening of psychiatric conditions has important implications for psychiatric prognostics and therapeutics. In our previous study, patients with prior psychiatric disease reported increased suicidal ideation (10). Understanding factors associated with psychiatric disease during a pandemic can help the patients, their family, and caregivers to screen and identify those at an increased risk of mental health crisis situations such as suicide attempts. Factors identified in this study including gender-based factors and prior exposure to trauma warrant further investigation to ensure that health systems can provide for the needs of a vulnerable population.

Previous research also shows increased gender-based disparity in association with humanitarian crises (35). During the Ebola outbreak of 2014–2016, women were reported to be at an increased risk of abuse, violence, and a lack of access to protective instructions (32). Moreover, women are more susceptible to the effects of economic insecurity, social isolation, disaster-related unrest, reduced health service accessibility, inability to escape abusive partners, and violence against healthcare workers. Measures such as social isolation have increased women's exposure to domestic violence: early reports from a police station in China's Hubei Province revealed three-times increased domestic violence targeting women during the COVID-19 quarantine period of February 2020 (32).

There are several strengths of our global and immediate approach to the examination of the vulnerable population of psychiatric patients during COVID-19. First, the sample size is large. Second, to reduce the participation bias, the study was administered in 11 different languages, ensuring generalizability across countries and cultures. Participants from the 12 countries represented a diverse perspective according to the economic structure and government support provided by their respective countries. For instance, countries such as Canada, France, Germany, Italy, Spain, Switzerland, and United States are classified as high-income economies according to the World Bank Atlas, whereas Bosnia and Herzegovina, Iran, Pakistan, and Turkey are considered middle- or lower-income countries (36). Third, as one of the earliest examinations of the mental health impact of COVID-19, our study carries the unique strength of immediate data collection during the peak of the COVID-19 pandemic in North America and Europe. In both the online survey and the clinical design, the psychiatric conditions were present in the patients at the baseline and thus indicate the potential effect of the pandemic on worsening of these conditions. Finally, the objective assessment of worsening of psychiatric conditions by the clinicians in the independent clinical cohort is an important contribution and strength of this study.

This study also has potential limitations that warrant consideration while interpreting the results. First, the sampling method is nonrandomized for the survey cohort. While a nonrandomized approach has potential disadvantages, we hope that the results of this study can nonetheless serve as a resource and catalyst for further investigation. For a similar global or continent-wide study, entities such as the WHO and the EU (European Union) could develop and administer a similar study with a wider reach. Second, the data were exclusively collected online for the survey—this may have excluded those less well-versed in web usage such as underdeveloped, rural, or disadvantaged populations. Nevertheless, to counter existing language barriers that may compound computer illiteracy, we translated the survey in native and official languages for each of the featured countries. Another important consideration is a potential confounding effect of other important factors, such as duration and severity of preexisting psychiatric conditions that may influence the impact of the current stressful events (37). Furthermore, quality of mental health services and mental health literacy could play an important role in mediating or modulating the effects of the pandemic on neuropsychiatric functioning of patients. Indeed, this could explain the regional differences in patient reports of psychiatric condition worsening. Lastly, a longitudinal assessment of the evolution of psychological symptoms in response to the COVID-19 pandemic is imperative and is the subject of an ongoing investigation by our group.

In conclusion, this effort highlights a significant impact of the COVID-19 pandemic on the mental health of psychiatric patients and elucidates prominent associations with their demographics, household conditions, personality traits, and attitude toward COVID-19. These results could serve to inform mental health professionals and policymakers across the globe, aiding in dynamic optimization of mental health services during and following the COVID-19 pandemic, and reducing its long-term morbidity and mortality.

## Data Availability Statement

The datasets generated in this study can be found in online repositories. The names of the repository/repositories and accession number(s) can be found below: All data presented in the main and Supplementary Items are deposited on the repository below and are available for verification upon request. https://osf.io/3vupe/?view_only=80f71b6f0c8d49b08573ea12eab10d33.

## Ethics Statement

The studies involving human participants were reviewed and approved by Zurich Cantonal Ethics Commission, Zurich, Switzerland Medical Faculty of the University of Tuzla, Tuzla, Bosnia Nencki Institute of Experimental Biology, Warsaw, Poland. The patients/participants provided their written informed consent to participate in this study.

## Author Contributions

SG and MP contributed in conceptualization, questionnaire development, data collection, data mining, data analysis, visualization, review, and editing. ZA contributed in data collection, manuscript writing, review, and editing. BS, SL, KA, AD, AB, LH, SE, HJ, LR-P, VW, AAl, MB, and DS contributed in questionnaire translation, data collection, data mining, review, editing, and project co-ordination. PR contributed in data analysis and visualization. RN contributed in data collection, manuscript writing, review, and editing. ZB-T contributed in data analysis. ZH, AAr, and SQ contributed in clinical data collection and project co-ordination. AJ contributed in conceptualization, questionnaire development, study approval, data collection, data analysis, data visualization, manuscript writing, review, editing, project administration, and supervision. All authors contributed to the article and approved the submitted version.

## Conflict of Interest

The authors declare that the research was conducted in the absence of any commercial or financial relationships that could be construed as a potential conflict of interest.
