# Supplementary material for: Worsening of Preexisting Psychiatric Conditions During the COVID-19 Pandemic
Source: Front Psychiatry. 2020 Dec 16;11:581426. doi: 10.3389/fpsyt.2020.581426 (PMC7772353; doi:10.3389/fpsyt.2020.581426)
Supplement: Supplementary file 1 [file Appendix_1.pdf]

Mental Health Impact of COVID-19

A risk and resilience study

Thank you for agreeing to participate in this survey on impact of COVID-19 on mental health. Your participation in this survey may aid health systems in dynamic optimization of mental health support for the masses during the existing or future crises. The questionnaire will take approximately 15 minutes of your time. Please make sure to answer all questions. The survey is completely anonymous and does not involve any risk of dissemination or misuse of your personal information. For any questions or feedback, please email the lead investigator Dr. Ali Jawaid on jawaid@hifo.uzh.ch.

Your participation in this research is voluntary. Your responses will be confidential and we do not collect identifying information such as name, email address or IP address. The results of this study will be used for scholarly purposes only and won't be disclosed or sold to third parties.

This survey has been approved and endorsed by the European MD-PhD Association, University of Zurich, Zurich Cantonal Ethics Commission, Nencki Institute of Experimental Biology, Poland and Medical Faculty of Tuzla, Bosnia.

1. Have you taken this survey before?

   Mark only one oval.

   - Yes
   - No

2. If yes, how long ago was it?

   Mark only one oval.

   - 0 - 3 days
   - 4 - 6 days
   - 7 - 13 days
   - > 14 days
   - Not applicable

3. what is your age? (Dropdown menu)

   ____

4. What is the gender you most identify with?

   Mark only one oval.

   - Female
   - Male
   - Non-binary/ Third gender
   - Prefer not to disclose

5. What country do you live in?

   Mark only one oval.

   (Dropdown menu)

6. What is your residential setting?

Mark only one oval.

- Urban
- Rural

7. What is your educational status?

Mark only one oval.

- Uneducated
- Primary School
- Secondary School
- Bachelors
- Masters degree
- Doctoral
- Post-doctoral

8. What is your current employment status?

Mark only one oval.

- Unemployed
- Employed (Public Sector)
- Employed (Private Sector)
- Self-employed/ Free-lancer
- Not applicable

9. Are you a medical/ healthcare professional in direct contact with COVID-19 patients?

Mark only one oval.

- Yes
- No

10. Are you currently working/ studying from home?

Mark only one oval.

- Yes
- No

11. Are you satisfied with the actions and attitude of your employer during the COVID - 19 pandemic?

Mark only one oval.

- Yes
- No
- Somewhat
- Not applicable

12. Are you satisfied with the response of your government with the COVID-19 pandemic?

Mark only one oval.

- Yes
- No
- Somewhat

13. Are you currently home-isolated?

Mark only one oval.

- Completely
- Largely
- Partially
- No

14. In case your answer to the previous question is completely or largely, please describe the status of your home isolation.

Mark only one oval.

- I am alone at home
- I am with a partner/friend
- I am with my family
- Not applicable

15. Do you have a pet at home?

Mark only one oval.

- Yes
- No

16. Are you able to interact with your family or friends during home isolation?

Mark only one oval.

- Yes, like usual
- Less than usual
- Minimal interaction

17. How would you rate your usage of social media tools during home isolation?

Mark only one oval.

- Like usual
- More than usual
- Less than usual

18. How much of your daily time are you devoting to physical exercise during home isolation?

Mark only one oval.

- 0 - 14 minutes
- 15 - 59 minutes
- 1 hour or more

19. Do you know a person close to you (co-worker, friend, or family member) who was found to be positive for COVID-19?

Mark only one oval.

- Yes
- No

20. Do you know a person close to you (co-worker, friend, or family member) who unfortunately died because of COVID-19?

Mark only one oval.

- Yes
- No

21. Do you have a previous history of a psychiatric illness?

Mark only one oval.

- Yes
- No

22. If yes, would you rate your condition being exacerbated by COVID-19/ home isolation?

Mark only one oval.

- Yes
- No
- Not applicable

23. Are you able to share your concerns and worries with a mental health professional these days?

Mark only one oval.

- Yes
- No
- Not applicable

24. Are you able to share your concerns and worries with your friends or family these days?

Mark only one oval.

- Yes, like usual
- Less than usual
- Not at all

25. Have you previously been exposed to a human crisis situation before (for example war, genocide, earthquake, flood)?

Mark only one oval.

- Yes
- No

26. Have you been exposed to a personal trauma (death of parent, parental separation, physical abuse, traumatic sexual experience, physical illness or injury requiring hospitalisation)?

Mark only one oval.

- Yes
- No

27. If yes, was it before or after the age of 17?

Mark only one oval.

- Before the age of 17
- After the age of 17
- I had traumatic experiences both before and after
- Not applicable

28. Would you describe yourself as an introvert or an extrovert?

Mark only one oval.

- Introvert
- Extrovert

29. Would you describe yourself as an optimistic or a pessimist?

Mark only one oval.

- Optimist
- Pessimist
- Neither of the above, I am a realist

30. What is your prediction about the current COVID-19 pandemic?

Mark only one oval.

- It will resolve within a month
- It will resolve in the summer but not within a month
- It will resolve after many months or years
- It might be the end of human race

31. Which one of the following statement matches (or is the closest) to the role you perceive for yourself in the current situation?

Mark only one oval.

- It is not in my control at all
- It is not in my control but I can take precautions to protect myself
- It is not in my control but I can take precautions to protect myself and also others

Please answer the following questions considering your living conditions in the past week.

32. In the current situation, do you often have headaches?

Mark only one oval.

- Yes
- No

33. Is your appetite poor?

Mark only one oval.

- Yes
- No

34. Do you sleep badly?

Mark only one oval.

- Yes
- No

35. Are you easily frightened?

Mark only one oval.

- Yes
- No

36. Do your hands shake?

Mark only one oval.

- Yes
- No

37. Do you feel nervous, tense or worried?

Mark only one oval.

- Yes
- No

38. Is your digestion poor?

Mark only one oval.

- Yes
- No

39. Do you have trouble thinking clearly?

Mark only one oval.

- Yes
- No

40. Do you feel unhappy?

Mark only one oval.

- Yes
- No

41. Do you cry more than usual?

Mark only one oval.

- Yes
- No

42. Do you find it difficult to enjoy your daily activities?

Mark only one oval.

- Yes
- No

43. Do you find it difficult to make decisions

Mark only one oval.

- Yes
- No

44. Is your daily work suffering?

Mark only one oval.

- Yes
- No

45. Are you unable to play a useful part in life?

Mark only one oval.

- Yes
- No

46. Have you lost interest in things?

Mark only one oval.

- Yes
- No

47. Do you feel that you are a worthless person?

Mark only one oval.

- Yes
- No

48. Has the thought of ending your life been on your mind?

Mark only one oval.

- Yes
- No

49. Do you feel tired all the time?

Mark only one oval.

- Yes
- No

50. Do you have uncomfortable feelings in your stomach?

Mark only one oval.

- Yes
- No

51. Are you easily tired?

Mark only one oval.

- Yes
- No

Below is a list of difficulties people sometimes have during stressful life events. Please read the item and indicate how distressing each difficulty is for you during the COVID-19 pandemic over the past week

0 - not at all 1 - a little bit 2 - moderately 3 - quite a bit 4 - extremely

52. Any reminder brings back feelings about the current crisis.

Mark only one oval.

	0	1	2	3	4	
not at all						extremely

53. I have trouble staying asleep.

Mark only one oval.

	0	1	2	3	4	
not at all						extremely

54. Other things keep making me think about the current crisis.

Mark only one oval.

	0	1	2	3	4	
not at all						extremely

55. I feel irritable and angry.

Mark only one oval.

	0	1	2	3	4	
not at all	⬭	⬭	⬭	⬭	⬭	extremely

56. I avoid letting myself get upset when I think about the current crisis or I am remindinding myself of it.

Mark only one oval.

	0	1	2	3	4	
not at all	⬭	⬭	⬭	⬭	⬭	extremely

57. I think about the current crisis when I am not meant to.

Mark only one oval.

	0	1	2	3	4	
not at all	⬭	⬭	⬭	⬭	⬭	extremely

58. I feel as if the current crisis isn't happening or isn't real.

Mark only one oval.

	0	1	2	3	4	
not at all	⬭	⬭	⬭	⬭	⬭	extremely

59. I stay away from reminders about the current crisis.

Mark only one oval.

	0	1	2	3	4	
not at all	⬭	⬭	⬭	⬭	⬭	extremely

60. Pictures about the current crisis pop into my mind.

Mark only one oval.

	0	1	2	3	4	
not at all	⬭	⬭	⬭	⬭	⬭	extremely

61. I am jumpy and easily startled.

Mark only one oval.

	0	1	2	3	4	
not at all	⬭	⬭	⬭	⬭	⬭	extremely

62. I try not to think about the current crisis

Mark only one oval.

	0	1	2	3	4	
not at all	⬭	⬭	⬭	⬭	⬭	extremely

63. I am aware that I still have a lot of feelings.

Mark only one oval.

	0	1	2	3	4	
not at all						extremely

64. My feelings about the current crisis are kind of numb.

Mark only one oval.

	0	1	2	3	4	
not at all						extremely

65. I have trouble falling asleep.

Mark only one oval.

	0	1	2	3	4	
not at all						extremely

66. I have waves of strong feelings about the current crisis

Mark only one oval.

	0	1	2	3	4	
not at all						extremely

67. I try to remove the current crisis from my memory.

Mark only one oval.

	0	1	2	3	4	
not at all						extremely

68. I have trouble concentrating.

Mark only one oval.

	0	1	2	3	4	
not at all						extremely

69. Reminders cause me to have physical reactions.

Mark only one oval.

	0	1	2	3	4	
not at all						extremely

70. I have dreams about the current crisis.

Mark only one oval.

	0	1	2	3	4	
not at all						extremely

71. I feel watchful and on guard.

Mark only one oval.

	0	1	2	3	4	
not at all	⬭	⬭	⬭	⬭	⬭	extremely

For each item 1 through 21, mark the severity 0, 1, 2, 3, which best describes your experience during COVID - 19 pandemic in the past week. The statements are arranged in an ascending order with 0 being the least severe and 3 being the most severe.

72. 1.

Mark only one oval.

- 0 - I do not feel sad.
- 1 - I feel sad.
- 2 - I am sad all the time and I can't snap out of it.
- 3 - I am so sad and unhappy that I can't stand it.

73. 2.

Mark only one oval.

- 0 - I am not particularly discouraged about the future.
- 1 - I feel discouraged about the future.
- 2 - I feel I have nothing to look forward to.
- 3 - I feel the future is hopeless and that things cannot improve.

74. 3.

Mark only one oval.

- 0 - I don't feel like a failure.
- 1 - I feel I have failed more than the average person.
- 2 - As I look back on my life, all I can see is a lot of failures.
- 3 - I feel I am a complete failure as a person.

75. 4.

Mark only one oval.

- 0 - I get as much satisfaction out of things as I used to.
- 1 - I don't enjoy things the way I used to.
- 2 - I don't get real satisfaction out of anything anymore.
- 3 - I am dissatisfied or bored with everything.

76. 5.

Mark only one oval.

- 0 - I don't feel particularly guilty.
- 1 - I feel guilty a good part of the time.
- 2 - I feel quite guilty most of the time.
- 3 - I feel guilty all of the time.

77. 6.

Mark only one oval.

- 0 - I dont feel I am being punished.
- 1 - I feel i may be punished.
- 2 - I expect to be punished.
- 3 - I feel I am being punished.

78. 7.

Mark only one oval.

- 0 - I don't feel disappointed in myself.
- 1 - I am disappointed in myself.
- 2 - I am disgusted with myself.
- 3 - I hate myself.

79. 8.

Mark only one oval.

- 0 - I don't feel I am any worse than anybody else.
- 1 - I am critical of myself for my weaknesses or mistakes.
- 2 - I blame myself all the time for my faults.
- 3 - I blame myself for everything bad that happens.

80. 9.

Mark only one oval.

- 0 - I don't have any thoughts of killing myself.
- 1 - I have thoughts of killing myself, but I would not carry them out.
- 2 - I would like to kill myself.
- 3 - I would kill myself if I had the chance.

81. 10.

Mark only one oval.

- 0 - I don't cry any more than usual.
- 1 - I cry more now than I used to.
- 2 - I cry all the time now.
- 3 - I used to be able to cry, but now I can't cry even though I want to.

82. 11.

Mark only one oval.

- 0 - I am no more irritated by things than I ever was.
- 1 - I am slightly more irritated now than usual.
- 2 - I am quite annoyed or irritated a good deal of the time.
- 3 - I feel irritated all the time.

83. 12.

Mark only one oval.

- 0 - I have not lost interest in other people.
- 1 - I am less interested in other people than I used to be.
- 2 - I have lost most of my interest in other people.
- 3 - I have lost all of my interest in other people.

84. 13.

Mark only one oval.

- 0 - I make decisions about as well as I ever could.
- 1 - I put off making decisions more than I used to.
- 2 - I have greater difficulty in making decisions more than I used to.
- 3 - I can't make decisions at all anymore.

85. 14.

Mark only one oval.

- 0 - I don't feel that I look any worse than I used to.
- 1 - I am worried that I am looking old or unattractive.
- 2 - I feel there are permanent changes in my appearance that make me look unattractive.
- 3 - I believe that I look ugly.

86. 15.

Mark only one oval.

- 0 - I can work about as well as before.
- 1 - It takes an extra effort to get started at doing something.
- 2 - I have to push myself very hard to do anything.
- 3 - I can't do any work at all.

87. 16.

Mark only one oval.

- 0 - I can sleep as well as usual.
- 1 - I don't sleep as well as I used to.
- 2 - I wake up 1-2 hours earlier than usual and find it hard to get back to sleep.
- 3 - I wake up several hours earlier than I used to and cannot get back to sleep.

88. 17.

Mark only one oval.

- 0 - My appetite is no worse than usual.
- 1 - My appetite is not as good as it used to be.
- 2 - My appetite is much worse now.
- 3 - I have no appetite at all anymore.

89. 18.

Mark only one oval.

- 0 - I haven't lost much weight, if any, lately.
- 1 - I have lost more than five pounds.
- 2 - I have lost more than ten pounds.
- 3 - I have lost more than fifteen pounds.

90. 19.

Mark only one oval.

- 0 - I am no more worried about my health than usual.
- 1 - I am worried about physical problems like aches, pains, upset stomach, or constipation.
- 2 - I am very worried about physical problems and it's hard to think of much else.
- 3 - I am so worried about my physical problems that I cannot think of anything else.

91. 20.

Mark only one oval.

- 0 - I don't get more tired than usual.
- 1 - I get tired more easily than i used to.
- 2 - I get tired from doing mostly anything.
- 3 - I am too tired to do anything.

92. 21.

Mark only one oval.

- 0 - I have not noticed any recent change in my desire for intimacy.
- 1 - I am less interested in the desire for intimacy than i used to.
- 2 - I have almost no desire for intimacy.
- 3 - I have lost the desire for intimacy completely.
- not applicable

Thank you for your time and effort

This content is neither created nor endorsed by Google.

Google Forms
