# Supplementary material for: Worsening of Preexisting Psychiatric Conditions During the COVID-19 Pandemic
Source: Front Psychiatry. 2020 Dec 16;11:581426. doi: 10.3389/fpsyt.2020.581426 (PMC7772353; doi:10.3389/fpsyt.2020.581426)
Supplement: Supplementary file 2 [file Appendix_2.pdf]

English questionnaire: https://forms.gle/Pcw4C2qz8H5GDZKW9

Arabic questionnaire: https://forms.gle/PRb226Uy1w9bTSMt8

Bosnian questionnaire: https://forms.gle/S5mWCghkBuWBUtGS8

French questionnaire: https://forms.gle/3qLGAnJ3dXkj5Njf7

German questionnaire: https://forms.gle/ZbraKp7LaLkTjh7N7

Greek questionnaire: https://forms.gle/f1JSsXprYY2SceaR8

Italian questionnaire: https://forms.gle/U2NYa9aS9d7rGYao9

Persian questionnaire: https://forms.gle/jTMsRdkuuZfChNeC9

Polish questionnaire: https://forms.gle/KqD8mkdRfMGNQTwn9

Spanish questionnaire: https://forms.gle/cN78PPbs88mhnTsq7

Turkish questionnaire: https://forms.gle/mpvHmN1cs663C4F47
