# Supplementary material for: Worsening of Preexisting Psychiatric Conditions During the COVID-19 Pandemic
Source: Front Psychiatry. 2020 Dec 16;11:581426. doi: 10.3389/fpsyt.2020.581426 (PMC7772353; doi:10.3389/fpsyt.2020.581426)
Supplement: Supplementary file 3 [file Data_Sheet_1.docx]

|  | n | Report of worsening |
| --- | --- | --- |
| PAHO | 56 | 27 (48.21%) |
| SEARO | 18 | 11 (61.11%) |
| EURO | 149 | 97 (65.10%) |
| EMRO | 52 | 26 (50.00%) |
| WPRO | 62 | 41 (66.12%) |
| Bosnia and Herzegovina | 58 | 26 (44.82%) |
| Canada | 246 | 199 (80.89%) |
| France | 50 | 24 (48.00%) |
| Germany | 123 | 67 (54.47%) |
| Iran | 132 | 74 (56.06%) |
| Italy | 88 | 43 (48.86%) |
| Pakistan | 203 | 147 (72.41%) |
| Poland | 255 | 137 (53.72%) |
| Spain | 205 | 112 (54.63%) |
| Switzerland | 79 | 40 (50.63%) |
| Turkey | 84 | 24 (28.57%) |
| United States | 874 | 590 (67.50%) |

**S1.** The number (n) and percentage of patients reporting worsening of their pre-existing psychiatric conditions.

| Psychiatric Condition | SRQ score | IES score | BDI score |
| --- | --- | --- | --- |
| No change | 6.2 ± 4.3 | 25.7 ± 13.1 | 10.6 ± 8.5 |
| Got worse | 12.5 ± 4.1 | 40.6 ± 12.8 | 22.5 ± 10.8 |

**S2. SRQ, IES, and BDI scores in the patients assessed.** Means and standard deviation for SRQ, IES and BDI scores in the patients assessed divided according to the status of their psychiatric condition during COVID-19 (no change vs. got worse).

|  |  | **N** | **%** |
| --- | --- | --- | --- |
| **Gender** | Female | 209 | 71.58 |
|  | Male | 83 | 28.42 |
| **Diagnosis** | Depression | 244 | 83.56 |
|  | Anxiety | 48 | 16.44 |
| **Social support during home isolation** | Yes | 46 | 15.81 |
|  | No | 246 | 84.19 |
| **Clinical identification of**  **new symptoms** | Other Symptoms | 6 | 2.05 |
|  | Suicidal | 1 | 0.34 |
|  | Psychosis | 2 | 0.68 |
|  | Aggression | 10 | 3.42 |
|  | Irritability | 11 | 7.19 |
|  | Sleep disturbances | 62 | 21.23 |
|  | Panic attacks | 14 | 4.79 |
|  | Substance abuse | 10 | 3.42 |
|  | Eating disturbance | 24 | 8.22 |
| **Clinical intervention** | Medication change | 54 | 18.49 |
|  | Medication adjusted | 35 | 11.99 |
|  | New Treatment | 51 | 20.89 |
|  |  |  |  |

**S3. Demographics of the clinical cohort.**
